# Virtual Reality Mindfulness Meditation for Patients After Anterior Cruciate Ligament Reconstruction: Protocol for the Development of a National Institutes of Health Stage 1 Behavioral Intervention

**DOI:** 10.2196/91897

**Published:** 2026-05-25

**Authors:** Shelby Baez, Hana Marmura, Brian Pietrosimone, Jason Moser

**Affiliations:** 1 Department of Exercise and Sport Science, College of Arts and Sciences, University of North Carolina at Chapel Hill, 312 Woollen Gymnasium, Chapel Hill, NC, 27514, United States, 1 919-445-1500, 1 919-445-1550; 2 Department of Psychology, College of Social Science, Michigan State University, East Lansing, MI, United States

**Keywords:** psychology, rehabilitation, knee injury, immersive therapy, meditation intervention, postoperative recovery, anterior cruciate ligament, ACL rehabilitation, reinjury prevention, sports injury psychology, psychological readiness, return-to-play confidence, movement biomechanics, landing mechanics, quadriceps function, stress regulation, present-moment awareness, feasibility protocol, pilot intervention, sham-controlled study, women in sport, anterior cruciate ligament reconstruction, post-ACLR care, behavioral rehabilitation, trial protocol

## Abstract

**Background:**

About 1 in 3 individuals will sustain a secondary anterior cruciate ligament (ACL) injury within 24 months of returning to sport after ACL reconstruction (ACLR). While aberrant biomechanics and poor quadriceps strength have been associated with secondary ACL injury risk, unresolved injury-related fear has also been identified as a potential risk factor for additional ACL injuries in previously high-functioning, physically active populations. Virtual reality mindfulness meditation (VRMM) is a psychological intervention that may reduce injury-related fear and improve an individual’s ability to stay in the present moment during stressful situations such as sport. There is a critical need to identify whether VRMM is a feasible intervention that could be implemented to address injury-related fear and reduce secondary ACL injury risk.

**Objective:**

This study aims to determine the feasibility, acceptability, and preliminary efficacy of a VRMM intervention with neuromuscular training rehabilitation (NTR) to reduce injury-related fear and improve jump-landing biomechanics in females with a history of ACLR, when compared to a sham virtual reality (VR) intervention+NTR.

**Methods:**

A double-blinded, 2-arm feasibility trial of 48 participants (24 per group) comparing VRMM+NTR and VR sham+NTR will be conducted. Recruitment, retention, adherence, and acceptability outcomes will be collected throughout the trial. Injury-related fear will be measured using the 11-Item Tampa Scale of Kinesiophobia. Jump-landing biomechanics will be assessed via peak knee abduction moment and peak knee flexion excursion. The change in outcomes will be compared between groups using 2×2 repeated measures ANOVA and partial η^2^ effect sizes. Significance will be set at *P*<.05.

**Results:**

The study was funded in May 2021. The trial started in September 2023 and is anticipated to be completed in May 2027, with the first results expected to be submitted for publication in winter 2027. We expect to observe acceptable rates of retention, adherence, and acceptability to the VRMM intervention and more favorable outcomes in the VRMM+NTR group compared to the VR sham+NTR group. As of now, 55 participants have been screened for eligibility, of which 33 were ineligible. Twelve participants have been randomized, and 10 have completed the trial. Two participants withdrew from the trial before the completion of the full intervention protocol due to the study time commitment.

**Conclusions:**

The results of this trial will improve our understanding of the feasibility of VRMM and inform the next steps in behavioral intervention development to test the efficacy of VRMM in reducing injury-related fear in females after ACLR.

## Introduction

Anterior cruciate ligament (ACL) injury is a traumatic knee injury that results in decreased quality of life, time loss from sport, and persistent knee symptoms, often leading to ACL reconstruction (ACLR) to restore knee stability [1]. ACLR helps patients return to previous levels of sports participation and engage in lifelong physical activity [1]. However, 1 out of 3 patients post-ACLR will not return to previous levels of sport participation, and injury-related fear is one of the primary barriers associated with failure to return to sport and decreased physical activity after ACLR [2-4]. Additionally, individuals with a history of ACLR who exhibit increased levels of injury-related fear also exhibit aberrant walking and jump-landing biomechanics after ACLR [5-7], with changes in jump-landing biomechanics being strongly associated with secondary ACL injury risk [8]. Supporting these associations, Paterno et al [9] identified that patients with increased injury-related fear after ACLR who return to sport are 13 times more likely to sustain a secondary ACL injury within 24 months when compared to those with lower levels of injury-related fear [9]. However, current rehabilitation programs often emphasize improvements in structural or biological aspects of the injury (eg, graft healing, neuromuscular training to address strength deficits, and improvement in range of motion) [10] and fail to address the psychological consequences of ACL injury and ACLR. As injury-related fear is associated with aberrant biomechanics and secondary ACL injury risk, there is a critical need to address injury-related fear to improve pertinent clinical outcomes after ACLR.

**Figure 1.**
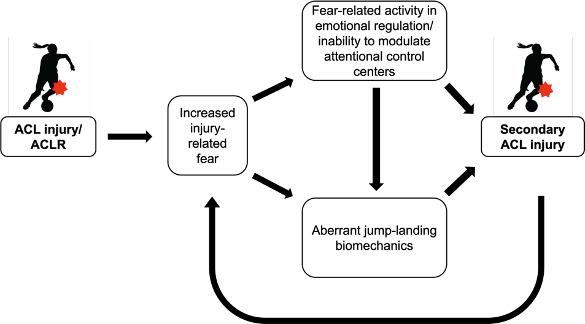
Hypothesized cycle of ACL injury, injury-related fear, and secondary ACL injury. ACL: anterior cruciate ligament; ACLR: anterior cruciate ligament reconstruction.

The stress and injury model is a theoretical model that may help to explain the relationship between injury-related fear and secondary ACL injury risk post-ACLR. This model hypothesizes that personality factors, history of stressors (eg, ACL injury), and coping strategies influence an individuals’ stress response (ie, cognitive appraisals, attentional demands, and physiological changes) and subsequent injury risk in an athletic context [11,12]. The stress and injury model proposes that encountering a stressful sports-specific situation (eg, a situation that mirrors the experience or mechanism of an individual’s ACL injury) may negatively affect an individual’s attentional control and lead to processing of irrelevant information [11,12]. During a heightened stress response, individuals may be unable to stay in the present and effectively attend to the situation, which may increase their risk to sustain a secondary ACL injury [13]. Attentional changes associated with injury-related fear may affect signaling from the central to the peripheral nervous system during sport-specific tasks, leading to cognitive-motor interference and altered knee joint biomechanics. For example, it has been observed that patients after ACLR exhibit changes in brain activity associated with attentional and emotional processing, including overactivity in the default mode network brain regions in females post ACLR [14-17]. Therefore, the cycle of ACL injury and secondary injuries may be perpetuated by increased injury-related fear, overactivity in emotional regulation, an inability to modulate attentional controls in brain regions (ie, default mode network), and altered jump-landing biomechanics (Figure 1).

The stress and injury model also proposes that interventions can be used to modify the stress response to reduce its impact when an athlete is faced with a stressful athletic situation. One intervention that may be helpful in addressing both emotion regulation and attentional control is mindfulness [18]. Mindfulness is a state of awareness of the present moment and allows for self-regulation of emotions and attention by decreasing rumination about past or future events [19]. Mindfulness can be cultivated through structural practices, such as mindfulness meditation, which encourages redirecting attention to a focal cue (eg, breath) to stay in the present moment [19]. During the practice of mindfulness, the individual is aware of all incoming thoughts and feelings, but rather than reacting to them, the individual accepts them and then continues to focus on the present moment, whether that is focusing on their breathing, movement, bodily sensations, or thoughts [19].

A positive association between mindfulness and psychological health and well-being has been identified [20-22]. Mindfulness interventions have led to the modulation of brain regions involved in emotional regulation and to improved attentional control in chronic pain and psychopathological populations [14-17], as well as reduced anxiety and fear in injured athletes, including patients with patellofemoral pain [23,24]. However, the effectiveness of mindfulness in improving functional outcomes, such as jump-landing biomechanics, has not been investigated in patients after ACLR. Improved emotional regulation and attentional control in sporting situations could improve biomechanics and decrease secondary ACL injury risk. Therefore, there is a critical need to explore whether mindfulness can improve clinically relevant outcomes in patients after ACLR.

Using established frameworks, such as the National Institutes of Health (NIH) stage model, can enhance our ability to generate and test interventions that can be implemented in real-world settings to address injury-related fear after ACLR. The NIH stage model consists of 6 different stages: basic science (stage 0); intervention generation, modification, refinement, adaptation, pilot, and feasibility testing (stage 1); traditional efficacy testing (stage 2); efficacy testing with real-world providers (stage 3); effectiveness research (stage 4); and dissemination and implementation research (stage 5) [25]. Stage 1 consists of all activities needed to create, test, and enhance the rigor of the behavioral intervention, including identifying implementation barriers from patients and providers. For example, it has been established that sports medicine providers are able to identify injury-related fear after a sport-related injury, but also report decreased confidence in implementing psychological skills and a lack of time for engaging in these approaches to address injury-related fear as common barriers [26]. Therefore, proposed interventions should consider solutions that will address these barriers to enhance the likelihood of implementation in real-world clinical settings.

Using technology-driven strategies may help reduce the associated implementation barriers for sports medicine providers in adopting psychologically informed approaches, such as mindfulness. The use of virtual reality (VR) can help to address these barriers by providing a plug-and-play solution while also enhancing the efficacy of mindfulness delivered to patients after ACLR. Furthermore, for novice meditators, VR can enhance the efficacy of mindfulness by engaging auditory and visual senses as attentional anchors to enhance the mindfulness experience [27-30]. Given the positive influence of VR mindfulness practices on outcomes in other clinical populations [27-30], and the opportunity to reduce known barriers to engaging in psychologically informed approaches in sport-related injury rehabilitation, there is a critical need to explore the efficacy of VR mindfulness practices in patients after ACLR. Furthermore, it is important to explore this intervention in a patient population most at risk for sustaining primary ACL injuries [31] and less likely to return to preinjury sport after ACLR [32] (ie, female athletes).

Female athletes are known to have higher sport dropout rates in adolescence, be at a greater risk of primary ACL injury, and may return to sport at lower rates following ACL injury for a multitude of reasons [31]. Additionally, female athletes post ACLR report differences in types of injury-related fear when compared to their male counterparts [33]. Consequently, when developing the proposed trial, we opted to recruit only female athletes post ACLR, as a fully powered study able to identify these nuances in sex differences would require substantially more resources than available. Therefore, the purpose of this study is to determine the feasibility (aim 1) and preliminary efficacy of an 8-week virtual reality mindfulness meditation (VRMM) intervention to decrease self-reported injury-related fear (aim 2a) and improve jump-landing biomechanics (aim 2b) in females cleared to return to activity post ACLR. We hypothesize that females cleared to return to activity post ACLR who undergo VRMM will demonstrate improvements in these surrogate end points associated with secondary ACL injury risk.

## Methods

### Study Design

A double-blinded, 2-arm feasibility trial will be conducted to investigate a VRMM intervention. To address aim 1, recruitment, retention, adherence, and acceptability outcomes will be collected throughout the trial. To address aim 2, changes in injury-related fear and jump-landing biomechanics will be compared between participants randomized to VRMM+neuromuscular training rehabilitation (NTR) or VR sham+NTR. The study schedule is outlined in Table 1.

**Table 1.** Schedule of events for the trial.

	Screening	Preassessment (wk 1)	Week 1 (3 visits)	Week 2 (3 visits)	Week 3 (3 visits)	Week 4 (3 visits)	Week 5 (3 visits)	Week 6 (3 visits)	Week 7 (3 visits)	Week 8 (3 visits)	Postassessment (wk 8)
Review eligibility	✓										
Informed consent		✓									
Demographics		✓									✓
Clinical history		✓									
Height and weight		✓									✓
Knee-related questionnaires IKDC^a^ Tegner ACL-RSI^b^		✓									✓
Injury-related fear (TSK-11)^c^	✓	✓									✓
Jump-landing biomechanics (LESS-RT)^d^	✓										
Jump-landing biomechanics (motion capture)		✓									✓
Adherence log			✓	✓	✓	✓	✓	✓	✓	✓	
Acceptability survey											✓
Randomization		✓									
Control and experimental intervention (neuromuscular training and virtual reality)			✓	✓	✓	✓	✓	✓	✓	✓	
Adverse event reporting		✓	✓	✓	✓	✓	✓	✓	✓	✓	✓

a IKDC: International Knee Documentation Committee.

b ACL-RSI: anterior cruciate ligament–return to sport after injury scale.

c TSK-11: 11-Item Tampa Scale of Kinesiophobia.

d LESS-RT: Landing Error Scoring System-Real Time.

### Sample Size Estimation and Study Population

For early-stage pilot and feasibility studies, it has been suggested to include at least 12 participants per group [34]. To achieve aim 1, a minimum of 12 participants in each group will be enrolled (ie, 24 participants). However, we also completed an *a priori power* calculation that determined a sample size of 20 participants per group (40 participants in total), which was required to achieve 80% power to detect a large between-group effect in injury-related fear. This calculation was based on a repeated measures ANOVA with a common SD of 1, a 2-sided α level of 0.05, and an estimated partial η^2^ of 0.23 [35] (moderate effect size) between the intervention and sham groups in injury-related fear. Therefore, a total of 48 participants (24 in each group) will be recruited to account for a 20% dropout rate in the sample and will be appropriate for making interpretations for aims 1 and 2.

### Recruitment and Eligibility

We will recruit from the student body at the University of North Carolina at Chapel Hill (UNC-CH), the UNC-CH Sports Medicine Institute, and through local community physical therapy clinic partners. All participants will review and sign a university-approved institutional review board informed assent/consent.

### Inclusion Criteria

Participants will be included based on the following inclusion criteria: (1) female sex assigned at birth, (2) age 14 to 35 years old, (3) 6 months to 10 years post ACLR, (4) ACL injury mechanism during sport activity (recreational or organized), (5) self-reported elevated injury-related fear defined as a score ≥17 on the 11-Item Tampa Scale of Kinesiophobia-11 [36] (TSK-11), (6) poor jump-landing movement quality defined as a score ≥5 on the Landing Error Scoring System-Real Time, and (7) cleared to return to unrestricted activity.

Females were selected for this study as female athletes are known to have higher sport dropout rates in adolescence, to be at a greater risk of primary ACL injury, to return to sport at lower rates following ACL injury for a multitude of reasons, and to exhibit differences in injury-related fear [31,37].

### Exclusion Criteria

Participants will be excluded if they (1) sustained a concussion or lower extremity injury in the last 3 months, (2) have any neurological conditions (ie, epilepsy) or are on any medications that affect the central nervous system, (3) had concomitant surgeries at the time of their index ACLR (eg, MCL repair), (4) are still actively participating in rehabilitation, or (5) are unable to attend intervention sessions 3 times per week.

### Randomization and Blinding

Participants will be randomized into either the VRMM+NTR group or the VR sham+NTR group, following the completion of preassessment outcome measurements (Table 1). A block randomization scheme will be used, and blocks ranging from 2 to 6 will be generated using an online randomization generator (allocation ratio 1:1). Group allocations will be sequentially numbered and sealed in opaque envelopes by a third-party individual not associated with the study. Interventionists, outcome assessors, and participants will be blinded to group allocation. After the collection of preassessment outcomes and prior to session 1, participants will be randomly assigned to their study condition (ie, VRMM+NTR group or VR sham+NTR group).

### Intervention and Sham Groups

#### Neuromuscular Training Rehabilitation

Both groups will complete a previously established, evidence-based late-phase progressive 8-week NTR (Table 2) [10]. This program is designed to address significant functional and neuromuscular limitations that are associated with ACL reinjury risk [10]. Four phases (2 weeks per phase; 3 times per week) will be used to appropriately progress participants through the program. Participants will be progressed through the following exercises: single-leg anterior bounding, lunges, lateral jumps, side planks, prone planks, posterior chain glute bridge, single-leg deadlifts, single-limb hop to stabilization, and single-leg stance (Table 2). These exercises do not directly address jump-landing biomechanics.

**Table 2.** Neuromuscular training rehabilitation (NTR) 8-week exercise progressions^a^.

Progression category	Phase 1 (Week 1 and 2)	Phase 2 (Week 3 and 4)	Phase 3 (Week 5 and 6)	Phase 4 (Week 7 and 8)
Single-leg anterior bounding	Double-leg takeoff to single-leg landing	Double-leg takeoff to single-leg bound	Single-leg takeoff to single-leg landing	Single-leg takeoff to single-leg bound
Rx^b^	3×10	3×10	3×10	3×10
Lunge	Static lunge	Dynamic lunge	Dynamic lunge with weight	Dynamic lunge with weight and trunk twist
Rx	3×10	2×30 feet	2×30 feet	20×30 feet
Lateral jump	Double-leg, noncontinuous	Double-leg, continuous	Single-leg, noncontinuous	Single-leg, continuous
Rx	2×10	2×10	2×10	2×10
Side plank	Bottom knee flexed	Bottom knee flexed+hip abduction	Legs extended	Legs extended+hip abduction
Rx	10×10 s	3×10	10×10–20 s	3×10
Prone plank	Forearms and knees	Hands and toes	Forearm and toes	Forearm and toes+leg lifts
Rx	10×10 s	10×10–30 s	10×10–30 s	3×10
Posterior chain glute bridge	Double-leg glute bridge on Bosu ball	Double-leg glute bridge on Bosu ball with leg extension	Double-leg glute bridge+roll out on the exercise ball	Double-leg glute bridge+leg lifts on the exercise ball
Rx	10×10 s	3×15	10×10 s	3×15
Single-leg deadlift	Flat surface, body weight	Flat surface with weight	Unstable surface, body weight	Unstable surface with weight
Rx	3×10	3×10	3×10	3×10
Single-limb hop to stabilization	18″ hop, arms out	18″ hop, hands on hips	27″ hop, arms out	27″ hop, hands on hips
Rx	10 A/P^c^, 10 M/L^d^	10 A/P, 10 M/L	1×10	1×10
Balance series	Tandem stance, eyes closed, arms across chest, firm surface	Single-leg stance, eyes closed, arms across chest, firm surface	Tandem stance, eyes closed, arms across chest, unstable surface	Single-leg stance, eyes closed, arms across chest, unstable surface
Rx	3×30 s	3×30 s	3×30 s	3×30 s

a For exercises involving a single-limb movement, the repetitions are for each side (ie, 3×15 double-leg bridge+leg lift on the ball would require 3 sets of 15 leg lifts on each side (30 in total).

b Rx: exercise prescription, displayed as sets×repetitions/duration.

c A/P: anterior/posterior.

d M/L: medial/lateral.

#### The VRMM Intervention

Participants randomized to the intervention group will undergo an 8-week VRMM intervention (3 times per week, 10-min sessions) in addition to the NTR program. VRMM engages visual and auditory senses to enhance the mindfulness experience and has been successfully used to address chronic pain [38] and anxiety [28,39]. The VRMM intervention will be administered with a commercially available wireless headset (MetaQuest, Meta Platform, Inc) using the Headspace XR application (Headspace, Inc). Prior to the first VRMM session, participants randomized into the VRMM+NTR group will watch a 10-minute educational video on the VR headset detailing the benefits of mindfulness. This will allow the participant to also gain exposure to the feel of the VR headset prior to the implementation of the intervention.

Participants enrolled in the VRMM group will complete a guided mindfulness experience that encourages observation, acceptance, and being present in their current environment through breath awareness. Participants will have the opportunity to complete a variety of mindfulness activities through the Headspace XR application, including focused attention mindfulness (eg, meditation and breath control) and mindful movement. While not directly associated with knee-specific activity, mindfulness was selected because individuals after ACLR must learn how to acknowledge their feelings while maintaining relaxation in specific environmental situations, rather than avoiding stressful situations or difficult emotions. Participants will also engage in mindful movement while in the headset to encourage mindfulness during the integration of movement. Engaging in mindfulness can be beneficial for retraining attentional control systems.

The Headspace XR application organizes its contents into three broad categories to align with different mindfulness goals and experiences. *Reflect* encourages present-moment awareness, introspection, and mental clarity. Tasks in this category will encourage the identification of current moods and reflection on current thoughts and feelings. *Unwind* promotes mind-body exercises aimed at releasing tension and enhancing breath awareness, such as diaphragmatic breathing. *Play* encourages mindfulness through movement and playful interactions to foster a mindful presence during activities. Across the duration of the 8-week intervention, participants will be advanced in their mindfulness practice to improve skills, such as focused attention and breathing awareness techniques (Table 3). Participants will complete the same, fixed-order combination of reflect, unwind, and play mindfulness activities throughout the 8-week intervention. Each full VRMM session is 10 minutes in length. Descriptions of included Headspace XR activities are presented in Table 4.

**Table 3.** Mindfulness intervention using MetaQuest virtual reality headset and Headspace XR application.

Weeks, session, and activity type	Name	Minutes (total=10 min)
1, 3, 5, 7		
1		
Reflect (mindfulness)	Mood stream	1
Unwind (mindfulness and mindful breathing)	Body scan	5
Play (mindful movement)	Focus slingshot	2
Reflect (mindfulness)	Take a beat meditation	2
2		
Reflect (mindfulness)	Mood stream	1
Unwind (mindfulness and mindful breathing)	Box breathing	2
Play (mindful movement)	Energy boost	5
Reflect (mindfulness)	Take a beat meditation	2
3		
Reflect (mindfulness)	Mood stream	1
Unwind (mindfulness and mindful breathing)	Kindness bubbles	5
Play (mindful movement)	Focus hoops	2
Reflect (mindfulness)	Take a beat meditation	2
2, 4, 6, 8		
1		
Reflect (mindfulness)	Mood stream	1
Unwind (mindfulness and mindful breathing)	Backward counting	5
Play (mindful movement)	Focus slingshot	2
Reflect (mindfulness)	Take a beat meditation	2
2		
Reflect (mindfulness)	Mood stream	1
Unwind (mindfulness and mindful breathing)	Body scan	5
Play (mindful movement)	Focus hoops	2
Reflect (mindfulness)	Take a beat meditation	2
3		
Reflect (mindfulness)	Mood stream	1
Unwind (mindfulness and mindful breathing)	Breathing slingshot	2
Play (mindful movement)	Energy boost	5
Reflect (mindfulness)	Take a beat meditation	2

**Table 4.** Headspace XR mindfulness activities description.

Activity name	Description
Mood stream	Players select daily mood and reflect on that emotion.
Body scan	A foundational full-body scan to notice sensations and reduce stress.
Focus slingshot	Players pay attention to timing to release the slingshot and paint a wall.
Take a beat meditation	Meditation based on selected mood.
Box breathing	Inhale 4 s, hold 4 s, exhale 4 s, hold 4 s guided by a visual.
Energy boost	Players collect orbs and stars through full-body movement.
Kindness bubbles	Players grab hearts representing positive feelings and pop bubbles to release negative ones.
Focus hoops	Players follow flashing hoops in sequence and shoot accordingly.
Backward counting	Players count backward while gently noticing distraction.
Breathing slingshot	Slingshot release timed with inhale and exhale.

The active ingredient of this intervention for participants is learning specific mindfulness skills and tools including focused attention, present-moment awareness, breathing skills, and mindful movement. It is hypothesized that these skills will provide participants with tools to overcome fearful situations, resulting in lower injury-related fear and improved jump-landing biomechanics following the intervention compared to the sham group.

#### VR Sham

Participants randomized to the sham group will undergo an 8-week VR sham (3 times per week, 10-min sessions) in addition to the NTR program. Participants in the sham group will be able to select and immerse themselves in VR environments (eg, beach and forest) using a medical-grade, wireless system (Limbix Health, Inc). However, participants in the sham group will not participate in any guided mindfulness meditation or mindful movement activities. While the sham environment may promote relaxation, these participants will not learn the mindfulness skills and techniques cultivated through the Headspace XR activities. Therefore, this group will receive an appropriately designed sham intervention by mimicking the experimental intervention as closely as possible, excluding only the active therapeutic element. It is hypothesized that without exposure to specific mindfulness tools, participants in the sham group will exhibit higher levels of injury-related fear and worse jump-landing biomechanics at the end of the study compared to the intervention group. Each VR sham session is 10 minutes in length.

### Treatment Fidelity

To ensure treatment fidelity, all interventionists will follow standard operating procedures that detail the VRMM+NTR procedures and the VR sham+NTR procedures, including the frequency, intensity, and duration of all activities. Additionally, prior to working with participants, all interventionists will undergo training to ensure that they are safely and effectively implementing the intervention and sham. Throughout the intervention, all interventionists will complete an adherence checklist to describe completed activities during each session. Finally, quality control checks will occur to identify issues as they arise and to maintain consistency of the intervention/sham delivery over time.

### Outcomes

#### Feasibility (Aim 1)

**Figure 2.**
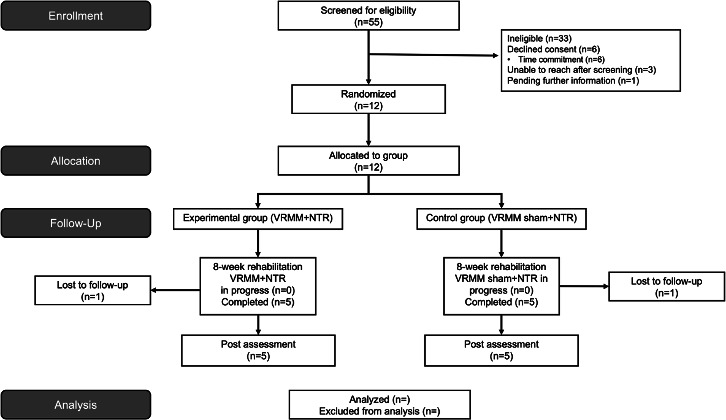
CONSORT (Consolidated Standards of Reporting Trials) participant flow diagram. NTR: neuromuscular training rehabilitation; VRMM: virtual reality mindfulness meditation.

Recruitment and retention (ie, screening, enrollment, consent, randomization, intervention/sham completion, and study completion) will be documented throughout the trial (Figure 2). Monthly recruitment rates will be calculated as the number of recruited and consented participants divided by the number of eligible participants (ie, those screened into the study). Regular enrollment reports outlining this information are being sent to the federal agency monthly. The goal recruitment rate is 1 to 3 participants per month over a 45-month timeframe (September 2023 to May 2027). Retention will be calculated as the percentage of participants retained during the 8-week period, and all reasons for dropout will be documented. We expect that more than 75% of participants will be retained during the 8-week period for both VRMM+NTR and VR sham+NTR groups.

Adherence to the VRMM and NTR components of interventions will be tracked by study interventionists. We expect moderate adherence (attending 50%‐75% of sessions) to high adherence (attending >75%) of sessions in 75% of participants throughout the 8-week trial. The type and number of mindfulness meditation sessions and the number of sets/reps of each exercise within the NTR program will be documented, along with any relevant notes about the sessions.

Acceptability will be assessed with a 6-item questionnaire that asks participants about the perceived helpfulness, benefits, and tolerability of both the VR and rehabilitation sessions (Table 5). Questions will be answered on a 0- to 100-point sliding visual analog scale with anchoring labels at 0, 50, and 100 (Table 4). The acceptability survey was adapted from previous works testing psychological interventions for patients following ACLR [40,41]. Participants in both the intervention and sham groups will answer the acceptability survey as both groups will be exposed to the NTR and VR headset in some capacity. We hypothesize that participants in the VRMM+NTR group will exhibit higher acceptability scores than those in the VRMM sham+NTR group.

**Table 5.** Acceptability questionnaire for participants, completed at the trial post assessment.

Question	Response anchors on visual analog scale
How helpful was the virtual reality?	Not helpful (0) Helpful (50) Most helpful (100)
How likely are you to recommend virtual reality to a friend?	Not likely (0) Likely (50) Most likely (100)
Please rate the benefit-to-effort relationship of the virtual reality.	The effort far outweighed the benefits (0) The effort equaled the benefits (50) The benefits far outweighed the effort (100)
How much did your fear change during the virtual reality?	Fear decreased a meaningful amount (0) Fear did not change (50) Fear increased a meaningful amount (100)
How tolerable was the virtual reality headset?	Not enjoyable (0) Neutral (50) Enjoyable (100)
How tolerable were the rehabilitation sessions?	Not enjoyable (0) Neutral (50) Enjoyable (100)

#### Injury-Related Fear (Aim 2a)

Injury-related fear will be measured by the TSK-11. The TSK-11 is a valid and reliable questionnaire that consists of 11 items scored on a 4-point Likert scale, which evaluates fear of movement and reinjury [36]. The score ranges from 11 to 44, with higher scores representing higher injury-related fear. The TSK-11 has been utilized in patients with ACLR [42], low back [36], upper extremity [36,43], and lower extremity disabilities [4]. Participants will complete the TSK-11 during screening to determine eligibility for the study based on elevated injury-related fear (>17). For participants consented into the trial, they will complete the TSK-11 at preassessment (wk 1), weeks 2, 4, and 6 of the intervention/sham, and the postassessment (wk 8) (Table 1).

#### Jump-Landing Biomechanics (Aim 2b)

Jump-landing biomechanics will be assessed by measuring peak knee abduction moments and knee flexion excursion during a drop vertical jump task with a 3D motion capture system. Participants will complete 3 successful trials of a drop vertical-jump assessment to evaluate external knee abduction moments and knee flexion excursion [44]. Participants will be instructed to drop directly down off a 30-cm box with 1 foot on each force plate and immediately perform a maximum vertical jump. Twenty-nine retroreflective markers will be placed bilaterally on the first and fifth metatarsal heads, medial and lateral malleoli, calcaneus, tibia, medial and lateral epicondyles, thigh, grater trochanter, anterior superior iliac spine, acromioclavicular joint, sternum, and L4/L5, and a cluster of 3 markers will be placed over the sacrum [44]. Prior to biomechanical testing, a static standing trial will be completed to assess lower extremity lengths between proximal and distal joint centers. Height of the center of mass will be calculated from the mass and inertial properties of each segment.

The 3D kinematic data will be sampled at 120 Hz using a 10-camera Bonita motion capture system (Vicon Motion Systems Ltd) and low-pass filtered at 10 Hz (fourth-order recursive Butterworth). Ground reaction forces will be collected at 1200 Hz using 2 calibrated force plates (AMTI OR-6) fixed in the floor and low-pass filtered at 75 Hz (fourth-order recursive Butterworth). Peak knee abduction moment will be calculated within the first 100 ms after initial contact of the foot on the force plate (ie, when vertical ground reaction force rises above 20 N), as this is when most noncontact ACL injuries occur [45]. Knee flexion excursion will be calculated during the landing phase (time between ground contact and peak knee flexion angle [46]). Inverse dynamics will be used to calculate moments and will be normalized to body weight and height (BW×m) [46]. Data will be analyzed using MATLAB (The MathWorks Inc) and LabVIEW (National Instruments).

### Statistical Analysis

Descriptive statistics (means, SD, frequency, and ranges) will be calculated for recruitment, retention rates, adherence, and acceptability outcomes. A threshold of 75% or better will be used as the minimum threshold for acceptable recruitment, adherence, and retention outcomes. A threshold of 50% will represent moderate acceptability, and scores of 80% or better will be used to identify high acceptability [40].

Descriptive statistics will be calculated for participant demographics, TSK-11 scores, peak knee abduction moment, and knee flexion excursion. A 2×2 repeated measures ANOVA will be used to assess changes between the intervention and sham groups. Group and time will serve as our independent variables. The TSK-11 score, peak knee abduction moment, and knee flexion excursion will serve as our dependent variables. The results will be reported as means and mean differences, with 95% CIs, *F*-values (ratio of between-group variance to within-group variance), and *P* values. The significance level will be set at a corrected value of *P*<.02 to reflect multiple comparisons (0.05/3). We hypothesize that patients in the VRMM+NTR group will demonstrate lower injury-related fear, decreased external knee abduction moment, and increased knee flexion excursion when compared to the VR sham+NTR group. Hypotheses for each outcome will be tested independently. We believe that these statistical differences will be supported with moderate to strong effect sizes. Partial η^2^ effect sizes will be calculated to examine the magnitude of the Group × Time interaction. Effect sizes will be interpreted as small if between 0.01 and 0.08, medium if between 0.09 and 0.24, and large if >0.25 [47].

### Ethical Considerations

This study obtained ethical approval from the UNC-CH institutional review board through the Office of Human Research Ethics (22‐0493). The study is funded by the National Institute of Arthritis and Musculoskeletal and Skin Diseases (K23AR079056-04). The trial was registered on ClinicalTrials.gov on September 2, 2022, and most recently updated on April 14, 2025 (NCT05527171). Recruitment began on September 5, 2023, and will be completed by approximately May 31, 2027.

Participants will be assigned a study ID to protect identifiable data (ie, VRMM_001 to VRMM_048). All signed consent forms will be locked in the principal investigator’s office, separate from all other study data. Study data will be stored in the REDCap (Research Electronic Data Capture) system, a secure web application provided by the North Carolina Translational and Clinical Sciences Institute for building and managing case report forms. Data and forms checking and double data entry will be conducted, with final data quality control performed by the principal investigator.

Adverse events will be recorded throughout the trial and will be reported in aggregate to the safety officer and National Institute of Arthritis and Musculoskeletal and Skin Diseases as part of routine data safety monitoring reports. Intentional unblinding will occur only for the participant’s safety when a serious adverse event occurs. The principal investigator and the safety officer will be notified of any serious adverse events.

## Results

The study was funded in May 2021. The trial started in September 2023 and is anticipated to be completed in May 2027, with first results expected to be submitted for publication in winter 2027. As of now, 55 participants have been screened for eligibility, of whom 33 were ineligible (Figure 2). Twelve participants have been randomized, and 10 have completed the trial (Figure 2). Two participants withdrew from the trial before the completion of the full intervention protocol due to the study time commitment.

## Discussion

### Rationale and Principal Findings

Psychological factors related to sports-related injuries and, specifically, outcomes after ACLR have recently garnered increased attention. Importantly, the influence of elevated injury-related fear has been linked to return-to-sport failures, decreased physical activity, and higher secondary injury rates [4,9,48]. Therefore, interventions are needed to address injury-related fear after ACLR. However, psychologically informed rehabilitation strategies for patients after ACL injury and reconstruction are still in their infancy. Systematic reviews have compiled a handful of articles investigating psychological interventions after ACLR, which include guided imagery and relaxation, web-based support, coping modeling videos, therapeutic insight videos, and neurocognitive rehabilitation approaches [49,50]. These reviews reveal a small amount of low-quality evidence, suggesting that psychological interventions can improve short-term outcomes (ie, self-reported injury-related fear and pain) compared to standard rehabilitation [49].

We propose a methodologically rigorous, low risk-of-bias study to address the concerns in the literature regarding the low-quality evidence supporting psychological interventions to improve outcomes after ACLR. This study will contribute to this growing area of research in psychological interventions to improve health outcomes after ACLR by investigating the use of VRMM in addition to standard neuromuscular rehabilitation in a randomized trial in patients after ACLR. While all participants will experience the VR headset and NTR, we expect to see superior results in patients who are exposed to the active ingredients: guided mindfulness including focused attention, breathing awareness techniques, and mindful movement.

Furthermore, we are following the NIH stage model’s systematic framework for behavioral intervention development to guide our study. This study is in stage 1 as we primarily aim to identify the feasibility of our intervention. However, we are optimistic that the results from this study will allow our research team to transition into stage 2 to evaluate the efficacy of the VRMM intervention in a traditional efficacy research study. We expect to move forward with the development of a larger-scale clinical trial if our hypotheses are supported regarding recruitment, retention, adherence, and acceptability and if promising preliminary efficacy for outcomes in aim 2 is observed (eg, Partial η^2^ ≥0.09). Following the procedures outlined in the NIH stage model will allow our team to develop a highly potent and maximally implementable behavioral intervention to improve clinical outcomes in patients after ACLR.

### Limitations and Anticipated Problems

This trial is designed to assess the feasibility and preliminary efficacy of VRMM in patients after ACLR. Therefore, we will not be able to draw any firm conclusions regarding the effectiveness of the VRMM intervention to improve the measured outcomes in this population. A larger randomized clinical trial will be needed to investigate the efficacy of VRMM+NTR compared to VR sham+NTR. The results from this trial will determine whether such a study is feasible and how a fully powered clinical trial may be optimally designed for this purpose.

Additionally, there are a total of 26 participant visits from preassessment to postassessment involved in this trial. Therefore, it is possible that participants may drop out, miss a study visit/visits, or that there may be missing data, which would introduce a risk of attrition bias. The feasibility outcomes are being used to understand these factors, and our sample size was calculated to account for possible attrition. Finally, the TSK-11 scale will be interpreted cautiously in view of the limitation that the scale was not developed specifically for acute musculoskeletal injuries; however, psychometric properties suggest that this scale can be applied to help understand outcomes after ACLR [42].

### Conclusion

To the best of our knowledge, this feasibility trial will be the first study to investigate the use of VRMM in the post-ACLR population. The effect of this intervention will be compared to a sham group, and both groups will simultaneously receive standard-of-care neuromuscular rehabilitation. Outcomes of interest include the feasibility of the trial, acceptability of the intervention, self-reported injury-related fear, and jump-landing biomechanics. The results of this trial will improve our understanding of how psychological interventions may be used to address injury-related fear in females after ACLR.

## Supplementary material

10.2196/91897 Peer Review Report 1 Peer-review report by AMS—Arthritis and Musculoskeletal and Skin Diseases Special Grants Review Committee, National Institute of Arthritis and Musculoskeletal and Skin Diseases (National Institutes of Health).

## Abbreviations:

ACL: anterior cruciate ligament
ACLR: anterior cruciate ligament reconstruction
NIH: National Institutes of Health
NTR: neuromuscular training rehabilitation
REDCap: Research Electronic Data Capture
TSK-11: 11-Item Tampa Scale of Kinesiophobia
UNC-CH: University of North Carolina at Chapel Hill
VR: virtual reality
VRMM: virtual reality mindfulness meditation

## References

[1] Adams D, Logerstedt DS, Hunter-Giordano A, Axe MJ, Snyder-Mackler L (2012). Current concepts for anterior cruciate ligament reconstruction: a criterion-based rehabilitation progression. J Orthop Sports Phys Ther.

[2] Ardern CL, Taylor NF, Feller JA, Webster KE (2014). Fifty-five per cent return to competitive sport following anterior cruciate ligament reconstruction surgery: an updated systematic review and meta-analysis including aspects of physical functioning and contextual factors. Br J Sports Med.

[3] Ross MD (2010). The relationship between functional levels and fear-avoidance beliefs following anterior cruciate ligament reconstruction. J Orthop Traumatol.

[4] Kvist J, Ek A, Sporrstedt K, Good L (2005). Fear of re-injury: a hindrance for returning to sports after anterior cruciate ligament reconstruction. Knee Surg Sports Traumatol Arthrosc.

[5] Baez S, Collins K, Harkey M (2023). Kinesiophobia is associated with peak knee abduction angle during jump landing after ACL reconstruction. Med Sci Sports Exerc.

[6] Trigsted SM, Cook DB, Pickett KA, Cadmus-Bertram L, Dunn WR, Bell DR (2018). Greater fear of reinjury is related to stiffened jump-landing biomechanics and muscle activation in women after ACL reconstruction. Knee Surg Sports Traumatol Arthrosc.

[7] Lisee C, Baez S, Bjornsen E (2025). Investigating the impact of preoperative kinesiophobia and pain on postoperative gait biomechanics following anterior cruciate ligament injury. Orthop J Sports Med.

[8] Paterno MV, Schmitt LC, Ford KR (2010). Biomechanical measures during landing and postural stability predict second anterior cruciate ligament injury after anterior cruciate ligament reconstruction and return to sport. Am J Sports Med.

[9] Paterno MV, Flynn K, Thomas S, Schmitt LC (2018). Self-reported fear predicts functional performance and second ACL injury after ACL reconstruction and return to sport: a pilot study. Sports Health.

[10] Di Stasi S, Myer GD, Hewett TE (2013). Neuromuscular training to target deficits associated with second anterior cruciate ligament injury. J Orthop Sports Phys Ther.

[11] Andersen MB, Williams JM (1988). A model of stress and athletic injury: prediction and prevention. J Sport Exercise Psychol.

[12] Andersen MB, Williams JM (1999). Athletic injury, psychosocial factors and perceptual changes during stress. J Sports Sci.

[13] Baez SE, Hoch JM, Cormier M (2020). The stress and injury model and cognitive appraisal model: implications for patients after anterior cruciate ligament reconstruction. Athl Train Sports Health Care.

[14] Berkovich-Ohana A, Glicksohn J, Goldstein A (2012). Mindfulness-induced changes in gamma band activity - implications for the default mode network, self-reference and attention. Clin Neurophysiol.

[15] Brewer JA, Worhunsky PD, Gray JR, Tang YY, Weber J, Kober H (2011). Meditation experience is associated with differences in default mode network activity and connectivity. Proc Natl Acad Sci U S A.

[16] Garrison KA, Zeffiro TA, Scheinost D, Constable RT, Brewer JA (2015). Meditation leads to reduced default mode network activity beyond an active task. Cogn Affect Behav Neurosci.

[17] Taylor VA, Daneault V, Grant J (2013). Impact of meditation training on the default mode network during a restful state. Soc Cogn Affect Neurosci.

[18] Tang YY, Hölzel BK, Posner MI (2015). The neuroscience of mindfulness meditation. Nat Rev Neurosci.

[19] Ludwig DS, Kabat-Zinn J (2008). Mindfulness in medicine. JAMA.

[20] Hölzel BK, Carmody J, Vangel M (2011). Mindfulness practice leads to increases in regional brain gray matter density. Psychiatry Res.

[21] King AP, Block SR, Sripada RK (2016). A pilot study of mindfulness-based exposure therapy in OEF/OIF combat veterans with PTSD: altered medial frontal cortex and amygdala responses in social-emotional processing. Front Psychiatry.

[22] Morone NE, Greco CM, Weiner DK (2008). Mindfulness meditation for the treatment of chronic low back pain in older adults: a randomized controlled pilot study. Pain.

[23] Mohammed WA, Pappous A, Sharma D (2018). Effect of mindfulness based stress reduction (MBSR) in increasing pain tolerance and improving the mental health of injured athletes. Front Psychol.

[24] Bagheri S, Naderi A, Mirali S, Calmeiro L, Brewer BW (2021). Adding mindfulness practice to exercise therapy for female recreational runners with patellofemoral pain: a randomized controlled trial. J Athl Train.

[25] Vranceanu AM, Kronish IM, Plys E, Syme M, Yu F, Gaugler JE (2025). The NIH stage model for behavioral interventions in aging: development, maturation, impact, and opportunities for advancement. Gerontologist.

[26] Cormier ML, Zizzi SJ (2015). Athletic trainers’ skills in identifying and managing athletes experiencing psychological distress. J Athl Train.

[27] Carl E, Stein AT, Levihn-Coon A (2019). Virtual reality exposure therapy for anxiety and related disorders: a meta-analysis of randomized controlled trials. J Anxiety Disord.

[28] Maples-Keller JL, Bunnell BE, Kim SJ, Rothbaum BO (2017). The use of virtual reality technology in the treatment of anxiety and other psychiatric disorders. Harv Rev Psychiatry.

[29] McCann RA, Armstrong CM, Skopp NA (2014). Virtual reality exposure therapy for the treatment of anxiety disorders: an evaluation of research quality. J Anxiety Disord.

[30] Opriş D, Pintea S, García-Palacios A, Botella C, Szamosközi Ş, David D (2012). Virtual reality exposure therapy in anxiety disorders: a quantitative meta-analysis. Depress Anxiety.

[31] Marmura H, Bryant DM, Getgood AM (2021). Infographic. Sex differences and ACL injuries. Br J Sports Med.

[32] Kuenze C, Lisee C, Pfeiffer KA (2019). Sex differences in physical activity engagement after ACL reconstruction. Phys Ther Sport.

[33] Lisee CM, DiSanti JS, Chan M (2020). Gender differences in psychological responses to recovery after anterior cruciate ligament reconstruction before return to sport. J Athl Train.

[34] Julious SA (2005). Sample size of 12 per group rule of thumb for a pilot study. Pharm Stat.

[35] Crescentini C, Chittaro L, Capurso V, Sioni R, Fabbro F (2016). Psychological and physiological responses to stressful situations in immersive virtual reality: differences between users who practice mindfulness meditation and controls. Comput Human Behav.

[36] Woby SR, Roach NK, Urmston M, Watson PJ (2005). Psychometric properties of the TSK-11: a shortened version of the Tampa Scale for Kinesiophobia. Pain.

[37] Arendt EA, Agel J, Dick R (1999). Anterior cruciate ligament injury patterns among collegiate men and women. J Athl Train.

[38] Gromala D, Tong X, Choo A, Karamnejad M, Shaw CD The virtual meditative walk: virtual reality therapy for chronic pain management.

[39] Navarro-Haro MV, López-Del-Hoyo Y, Campos D (2017). Meditation experts try virtual reality mindfulness: a pilot study evaluation of the feasibility and acceptability of virtual reality to facilitate mindfulness practice in people attending a mindfulness conference. PLoS One.

[40] Coronado RA, Sterling EK, Fenster DE (2020). Cognitive-behavioral-based physical therapy to enhance return to sport after anterior cruciate ligament reconstruction: an open pilot study. Phys Ther Sport.

[41] Baez S, Genoese F, Reiche E (2023). Feasibility of mobile application-delivered mindfulness meditation for individuals after anterior cruciate ligament reconstruction. Int J Athl Ther Train.

[42] George SZ, Lentz TA, Zeppieri G, Lee D, Chmielewski TL (2012). Analysis of shortened versions of the Tampa Scale for Kinesiophobia and Pain Catastrophizing Scale for patients after anterior cruciate ligament reconstruction. Clin J Pain.

[43] Bot SD, van der Waal JM, Terwee CB (2005). Predictors of outcome in neck and shoulder symptoms: a cohort study in general practice. Spine (Phila Pa 1976).

[44] Myer GD, Ford KR, Foss KDB, Rauh MJ, Paterno MV, Hewett TE (2014). A predictive model to estimate knee-abduction moment: implications for development of a clinically applicable patellofemoral pain screening tool in female athletes. J Athl Train.

[45] Hewett TE, Myer GD, Ford KR, Paterno MV, Quatman CE (2016). Mechanisms, prediction, and prevention of ACL injuries: cut risk with three sharpened and validated tools. J Orthop Res.

[46] Pfeiffer SJ, Blackburn JT, Luc-Harkey B (2018). Peak knee biomechanics and limb symmetry following unilateral anterior cruciate ligament reconstruction: associations of walking gait and jump-landing outcomes. Clin Biomech (Bristol).

[47] Cohen J (1973). Eta-squared and partial eta-squared in fixed factor ANOVA designs. Educ Psychol Meas.

[48] McPherson AL, Feller JA, Hewett TE, Webster KE (2019). Psychological readiness to return to sport is associated with second anterior cruciate ligament injuries. Am J Sports Med.

[49] Isaji Y, Uchino S, Inada R, Saito H (2024). Effectiveness of psychological intervention following anterior cruciate ligament reconstruction: a systematic review and meta-analysis. Phys Ther Sport.

[50] Coronado RA, Bird ML, Van Hoy EE, Huston LJ, Spindler KP, Archer KR (2018). Do psychosocial interventions improve rehabilitation outcomes after anterior cruciate ligament reconstruction? A systematic review. Clin Rehabil.

