# Supplementary material for: Virtual Reality Mindfulness Meditation for Patients After Anterior Cruciate Ligament Reconstruction: Protocol for the Development of a National Institutes of Health Stage 1 Behavioral Intervention
Source: JMIR Res Protoc. 2026 May 25;15:e91897. doi: 10.2196/91897 (PMC13200807; doi:10.2196/91897)
Supplement: Peer Review Report 1 [file resprot-v15-e91897-s001.pdf]

SUMMARY STATEMENT
(Privileged Communication)

PROGRAM CONTACT:
CHARLES WASHABAUGH
301-496-9568
washabac@mail.nih.gov

Release Date: **11/23/2020**
Revised Date:

Application Number: **1 K23 AR079056-01**

Principal Investigator

BAEZ, SHELBY

Applicant Organization: MICHIGAN STATE UNIVERSITY

Review Group: **AMS**
Arthritis and Musculoskeletal and Skin Diseases Special Grants Review Committee

Meeting Date: **10/22/2020**
Council: **JAN 2021**
Requested Start: **05/01/2021**
RFA/PA: **PA20-206**
PCC: **3 B**
Dual IC(s): **HD**

Project Title: **Virtual Reality Mindfulness Meditation in Patients after Anterior Cruciate Ligament Reconstruction**
SRG Action: **Impact Score:24**
Next Steps: **Visit https://grants.nih.gov/grants/next_steps.htm**
Human Subjects: 48-At time of award, restrictions will apply
Animal Subjects: 10-No live vertebrate animals involved for competing appl.
Gender: 2A-Only women, scientifically acceptable
Minority: 1A-Minorities and non-minorities, scientifically acceptable
Age: 6A-Children and Adults, scientifically acceptable

Project Year	Direct Costs Requested	Estimated Total Cost
1	121,418	130,983
2	133,321	143,823
3	137,932	148,798
4	137,795	148,650
5	132,237	142,654
TOTAL	662,703	714,908

ADMINISTRATIVE BUDGET NOTE: The budget shown is the requested budget and has not been adjusted to reflect any recommendations made by reviewers. If an award is planned, the costs will be calculated by Institute grants management staff based on the recommendations outlined below in the COMMITTEE BUDGET RECOMMENDATIONS section.

1K23AR079056-01 BAEZ, SHELBY

PROTECTION OF HUMAN SUBJECTS UNACCEPTABLE

RESUME AND SUMMARY OF DISCUSSION: This is a new application for a K23 Mentored Patient-Oriented Research Career Development Award from Shelby Baez, PhD at the Michigan State University (MSU). Dr. Baez proposes to conduct a small-scale clinical trial in women 9-months post-ACL reconstruction (ACLR) to study the effect of virtual reality mindfulness meditation (VRMM) on injury prevention through reducing fear, correcting jump-landing patterns and improving brain activity. The candidate is viewed as outstanding. Dr. Baez has a strong background in Exercise Science and is at the appropriate career stage for the proposed training. The reference letters are highly supportive. There is a concern about the candidate's relatively low number of first author research-based publications, but this is considered minor. The career development plan is well organized and focused, although it might be further strengthened by more training in mindfulness meditation, behavioral intervention and clinical trial methodology. Another strength is the outstanding research environment at the MSU and the strong institutional support for the candidate. The research plan is very well presented, highly innovative, feasible and viewed as an excellent training vehicle for the candidate, despite some questions about the trial design. Dr. Matthew Pontifex is considered an outstanding mentor for providing the expertise in training and will be complemented by Drs. Brian Pietrosimone and Taosheng Liu. However, the relative paucity of research support among the mentors is a concern. Overall, strengths outweigh weaknesses and the committee remains enthusiastic about the independent career developmental potential for this candidate.

DESCRIPTION (provided by applicant): Injury-related fear after anterior cruciate ligament (ACL) injury significantly contributes to decreased return to sport, decreased physical activity engagement, and increased ACL reinjury risk in previously high functioning, physically active individuals. Injury-related fear is also associated with poor jump-landing movement patterns in patients after ACL reconstruction (ACLR). Unfortunately, current rehabilitation practices do not specifically address injury-related fear in patients after ACLR. Virtual reality mindfulness meditation is a mental practice that involves focusing your mind on your experiences in the present moment and has been used to address depression, anxiety, and chronic musculoskeletal pain. Additionally, mindfulness meditation has led to positive changes in brain activity in individuals with depression, anxiety, and chronic musculoskeletal pain. Patients after ACLR exhibit poor brain activity similar to individuals with depression, anxiety, and chronic musculoskeletal pain; thus, patients after ACLR may also benefit from mindfulness meditation. To evaluate the effect of virtual reality mindfulness meditation on patients after ACLR, we will determine the effect of virtual reality mindfulness meditation to 1) decrease self-reported injury-related fear, 2) improve poor jump-landing movement patterns, and 3) improve brain activity in women 9-months post-ACLR when compared to a virtual reality sham group. Injury-related fear and poor jump-landing movement patterns have been associated with ACL reinjury risk. Women were selected for this study as previous literature has demonstrated sex differences in brain activity and women also have a higher incidence of ACL injury and ACL reinjury risk. All patients in the study will complete an 8-week advanced rehabilitation training program to improve lower-body muscle strength, power, and flexibility. Patients in the intervention group will complete 8-weeks of virtual reality mindfulness meditation + the advanced rehabilitation training program. Patients in the sham group will complete 8-weeks of the virtual reality sham + the advanced rehabilitation training program. The central hypothesis is that women 9-months post ACLR who undergo virtual reality mindfulness meditation will demonstrate decreased injury-related fear, improved jump-landing movement patterns, and improved brain activity. The findings from this proposal will address a gap in knowledge about effective psychological interventions to address psychological and biological factors associated with ACL reinjury risk. This proposal also positions an aspiring independent clinician scientist training to learn neuroimaging techniques and complex movement pattern data collection and analyses. With this training, the primary investigator will examine the efficacy of virtual reality mindfulness meditation to mitigate injury-related

fear, decrease ACL reinjury risk, and improve quality of life in patients after ACLR throughout her career as a clinician scientist.

PUBLIC HEALTH RELEVANCE: Eliminating injury-related fear in patients after ACL reconstruction can decrease the healthcare burden associated with ACL reinjury rates by allowing patients to safely return to physical activity, improve their overall health and wellness, and decrease the risk for the development of overuse conditions like post-traumatic knee osteoarthritis. Relevant to the NIH mission, virtual reality mindfulness meditation may incite a paradigm shift in current treatment strategies, by promoting patient-centered holistic healthcare, for the hundreds of thousands of individuals who sustain an ACL injury and undergo ACL reconstruction.

CRITIQUE 1

Candidate: 2
Career Development Plan/Career Goals: 3
Research Plan: 3
Mentor(s), Co-Mentor(s), Consultant(s), Collaborator(s): 4
Environment Commitment to the Candidate: 2

Overall Impact: This is a very interesting K23 application for mentored training in patient-oriented research from Dr. Baez, an Assistant Professor of Kinesiology at Michigan State University. The candidate has strong academic training in exercise science and exposure to neuroimaging techniques. The currently proposed project and career development plan is a novel integration of her background in athletic training and kinesiology with neuroscience and behavioral interventions. The proposal is very well written and it is clear that the candidate has very strong potential and high motivation to become an independently funded investigator. The research topic, i.e., using virtual reality mindfulness meditation along with neuromuscular exercise training to reduce fear of re-injury in young women who are in rehab therapy after surgical ACL repair, is very significant and potentially impactful in the field. Her mentors have strong content expertise and the institutional commitment is excellent. However, the main concerns about this application are the relative weakness in training with regard to implementation of rigorous research, increasing knowledge about mindfulness techniques and the mentors' limited track record of independent (NIH R01 level) funding. The small-scale clinical trial proposed is somewhat underdeveloped and needs further input from experts in clinical trial methodology so that the candidate will be able to use this mentored award to prepare for larger intervention studies in the future as they propose.

1. Candidate:
Strengths

- Dr. Baez is a first-year Assistant Professor of Kinesiology at Michigan State University. She has a background in Exercise and Sports Science with a PhD in Rehabilitation Sciences. She has strong academic training that is well-aligned with the goals for career development and research in this K23 application. She is at the right career stage where this type of mentored award could be very useful in launching her independent career.
- The candidate has been quite productive with 14 peer-reviewed publications (5 first author). She also has obtained some seed grant funding through institutional resources and a number of grants/scholarships from Athletic Trainer's associations.
- Letters are strong and attest to her high potential to become an independent researcher in the unique intersection between exercise science, psychological interventions and neuroscience in the field of injury prevention.

Weaknesses

- Publications are primarily in specialty sports medicine journals and first author papers are mainly reviews and few data-based studies.
- So far it does not appear that her training has included exposure to working in large NIH funded labs although her career goals are to become an R01 level funded investigator.

2. Career Development Plan/Career Goals & Objectives:

Strengths

- The content of proposed mentored training is well-suited to the candidate's interests and well-described in the proposal.
- The candidate has strong experience working clinically with young athletes with ACL injuries and has good knowledge about psychological factors such as fear of re-injury being related to altered biomechanics such that re-injury becomes more likely.
- The career development plan was impressively focused and well thought out.
- The gaps in training she has identified are mainly in the areas of 1) biomechanics assessment and analysis, 2) neuroimaging techniques and analysis and 3) quantitative methods. The training she will receive in each of these areas is well articulated and will progress from mentored analysis of existing datasets, experiential training in techniques and finally, hands on training through her proposed small scale trial of virtual reality mindfulness to improve fear of re-injury/movement and jump landing performance alterations (which are a known risk for re-injury).

Weaknesses

- The candidate does not address whether any additional training in behavioral interventions – particularly in mindfulness meditation (with or without VR delivery) will be included.
- Stronger training in clinical trials methodology and implementation would strengthen the career development plan.

3. Research Plan:

Strengths

- The topic of the research plan is highly significant with a focus on prevention of re-injury in female athletes who have undergone surgery for ACL repair, an extremely common sports injury particularly among young women. A third of these women do not return to sports/high levels of physical activity because of fear of re-injury. There is some literature that suggests that fear of re-injury is associated with alterations in biomechanics which in fact makes the person more prone to injuries. There are also studies that suggest neural changes – lower ability to inhibit default mode network (DMN) activity in the brain are associated with fear/anxiety.
- The candidate is proposing a conceptually well-grounded research program that brings together various fields to conduct a small clinical trial to examine whether a psychological technique (Virtual Reality Mindfulness Meditation; VRMM) can reduce fear of movement and improve biomechanics in adolescents and young women (ages 14-25, the prime age for these injuries) undergoing rehabilitative training 9 months post ACLR. A third aim will test whether DMN suppression is superior in the group that receives VRMM using an experimental task in the fMRI scanner.

- The area of research is seen as highly innovative and technologically sophisticated. Her mentors appear to have the expertise, equipment and facilities to implement the novel study proposed.
- The study is generally well-described and feasible in the time frame of this award. It will form an excellent platform for her training and career development.

Weaknesses

- As proposed, it is doubtful that the small-scale trial will provide sufficient evidence to launch a full-scale trial application by the end of the award.
- The candidate is proposing an efficacy study which is likely to be underpowered. Both groups are receiving 8 weeks of progressive neuromuscular training which is highly focused on improving strength, gait, posture etc. So regardless of VRMM, there is likely to be some effect on fear of injury and in particular, jump landing performance which relies on posterior chain muscles which will likely improve with exercise training alone. Since both groups are expected to improve to some extent - with the expectation that the VRMM group will show relatively greater improvement, a much larger sample size than 20 per group will be needed. A preliminary feasibility trial would likely be more appropriate at this stage of research on the novel combined approach of exercise + mindfulness training.
- Many details about clinical trial implementation (how a double blind will be maintained etc.) are not carefully thought through or explained.
- It is unclear if the VR headset and software are fully operational and have been piloted in this population to ensure engagement and developmental appropriateness for their age range.
- The experimental task in the scanner is very interesting but potentially investigated as a mediator of treatment effects (consistent with their conceptual model) rather than an outcome in and of itself. This will require a more sophisticated statistical plan.
- There are no measures of pain which is an oversight because movement-related pain could be an important driver of fear of movement/re-injury and should at least be measured.
- Overall, some input from experienced trialists in the planning of the study design and methods would be beneficial.

4. Mentor(s), Co-Mentor(s), Consultant(s), Collaborator(s):

Strengths

- The primary mentor will be Dr. Pontifex, an Associate Professor in the Department of Kinesiology. Dr. Pontifex has expertise in how physical activity and exercise impact cognitive function. His interests in cognitive neuroscience and physical exercise are a good fit with the candidate’s interests. He has a strong record of publications and experience mentoring doctoral and undergraduate students.
- The two secondary mentors have the expertise to provide the candidate with the technical training she proposes –in neuroimaging (Dr. Liu at MSU) and biomechanics methods and analysis (Dr. Pietrosimone at UNC-Chapel Hill). While Dr. Pietrosimone is not local, he has an ongoing mentoring relationship with the candidate through the national athletic trainers association.

Weaknesses

- Mentors, other than Dr. Liu who had an R01 which ended in 2018, have funding mostly from smaller (R21/R03) grants and a number of industry sources. It is unclear if they can provide the

level of training in grantsmanship that the candidate will require to become an independently (R01 level) funded investigator.

- Similarly, mentors' experience in RCTs is limited to relatively small-scale or collaborative studies. Stronger mentorship in trial design and implementation would be needed.
- Mindfulness meditation is a big focus of the research plan and additional professional development or mentorship in this content area would strengthen the application.

5. Environment and Institutional Commitment to the Candidate:
Strengths

- Michigan State University is a strong research institution and has excellent resources for career development of the candidate.
- Although there isn't a large medical center associated with MSU, their Sports Medicine clinic sees a large number of surgical ACL repair patients that would be eligible for the study so it would be feasible to implement.
- The Department is very supportive of the applicant's research career and will ensure she has the protected time to pursue the research and career development plan. She has a full-time faculty appointment and separate space will be allocated for her lab.

Weaknesses

- Local experience in large scale clinical trials seems very limited and it is unclear if the infrastructure to support this project and particularly, the future planned research will be adequate.

Study Timeline

Strengths

- A small-scale trial is proposed and seems quite feasible in the time-frame proposed.

Weaknesses

- None noted

Protections for Human Subjects

Unacceptable Risks and/or Inadequate Protections

- The research plan includes children under the age of 18 and parental informed consent will be needed for those participants. None is described.

Data and Safety Monitoring Plan (Applicable for Clinical Trials Only):

Unacceptable

- The study is described as a double-blind trial and yet safety monitoring will be done by the PI. It is unclear how the PI can be blinded and also monitor safety - particularly if any intervention-related adverse event were to occur.

Inclusion Plans

- Sex/Gender: Distribution justified scientifically
- Race/Ethnicity: Distribution justified scientifically
- For NIH-Defined Phase III trials, Plans for valid design and analysis: Not applicable

- Inclusion/Exclusion Based on Age: Distribution justified scientifically
- The study will include women only which is scientifically justified given that this particular sports injury is very common in women and the neuroimaging and biomechanics implications would be expected to be different for men. They would not have statistical power to test sex-based differences in this pilot study. All races/ethnicities would be eligible, and the representation is expected to be similar to the population of the geographic region.

Vertebrate Animals

Not Applicable (No Vertebrate Animals)

Biohazards

Not Applicable (No Biohazards)

Training in the Responsible Conduct of Research

Acceptable

Comments on Format (Required):

- Acceptable

Comments on Subject Matter (Required):

- Acceptable

Comments on Faculty Participation (Required; not applicable for mid- and senior-career awards):

- Acceptable

Comments on Duration (Required):

- Acceptable

Comments on Frequency (Required):

- Acceptable

Resource Sharing Plans

Acceptable

Authentication of Key Biological and/or Chemical Resources

Not Applicable (No Relevant Resources)

Budget and Period of Support

Recommend as Requested

CRITIQUE 2

Candidate: 1
Career Development Plan/Career Goals: 1
Research Plan: 3
Mentor(s), Co-Mentor(s), Consultant(s), Collaborator(s): 3

Environment Commitment to the Candidate: 1

Overall Impact: The application is an original K23 mentored clinical research application from a young Assistant Professor focused on the role of fear and neuropathic pain in recovery from ACL rupture. The PI is an Athletic Trainer with experience and training in psychological evaluation of athletes for fear following injury. The goal of the K23 is to become an expert in biomechanical analysis of knee/jumping mechanics and functional MRI based measures of brain activity. To achieve this goal, the PI has recruited two very strong mentors, one at her home University and the other a national expert a thousand miles away. The distance is not a concern as the two have been meeting by teleconference, have a mentorship relationship fostered by support from the NATA, and the PI plans 5 one-week intensive visits to the mentor's lab over the course of the award. This is very well written proposal with clear goals and outcome measures. The number of subjects is relatively low (48 over three years), but this reflects a realistic understanding of how far the grant support will go. The institution is providing teaching relief and a full-time graduate student to support the project. The one concern is that the primary mentor, though intellectually a good match for the research, does not have a track record of NIH R01 level funding or examples of successful launch committees that they have led. The addition of a strong NIH funded clinical researcher would be a great plus to the development of this strong young PI.

1. Candidate:
Strengths

- The PI is a PhD athletic trainer. She completed her PhD in two years finishing in 2019. The biosketch says that she was simultaneously working as an Assistant Professor (2017) at Michigan State, but I think that is a typo and 2017 should be 2019, indicating that she went directly from her PhD research into a faculty position and has had that position for one year.
- The PI has 14 peer-reviewed publications (5 first author) and a seed grant to start her research program.
- The letters are glowing in their support of the PI as an outstanding young clinician scientist.
- The PI's organizational skills are clear from the proposal. I have never seen a more thorough and detailed budget justification, right down to the journal fees for the target journals of the proposed manuscripts.
- This is an outstanding candidate.

Weaknesses

- None noted

2. Career Development Plan/Career Goals & Objectives:
Strengths

- The career development plan is exceptionally organized, clear and complete. There is no doubt that the PI will achieve her goals if she is able to complete all of the aspects of the training program described.
- Love the R-ladies group
- The coursework makes sense and has a clear purpose.

Weaknesses

- Averaging 3 national/international meetings a year might be a little much at the beginning, but this will be great for networking.

3. Research Plan:
Strengths

- The proposed work is novel and most importantly will provide the PI with training in biomechanics and neuroimaging that will allow her to develop a level of independence for her future studies on neurocognitive limits to ACL rehabilitation.
- The proof of concept data is provocative and provides a strong intellectual basis for the project.
- The idea of mindful meditation using VR is intellectually innovative
- The proposal is very manageable but will still provide insight into a potentially inexpensive way to improve return to play following injury.

Weaknesses

- None noted

4. Mentor(s), Co-Mentor(s), Consultant(s), Collaborator(s):
Strengths

- Dr. Pontifex will serve as primary mentor for the PI. His research focuses on changes in higher-order cognitive function as a result of activity.
- Dr. Liu is at MSU and contributes expertise in fMRI to allow the imaging of brain activity following the training intervention.
- Dr. Pietrosimone works out of UNC Chapel Hill and has expertise as a biomechanist. This expertise will be used to assist with the jump mechanics aspect of the proposal.

Weaknesses

- The primary mentor, who is charged with helping the PI generate R01 level funding has never held an R01 and is currently funded by an undisclosed company project. Even though Dr. Pontifex might be a great research mentor based on intellectual overlap, the primary mentor in a K23 should have a better funding record.

5. Environment and Institutional Commitment to the Candidate:
Strengths

- The Resources and facilities section are wonderful. It is clear from reading these two pages that the PI will have everything that she needs to complete the study.
- The fact that the University is providing Dr. Baez release from teaching and access to a fully funded doctoral research assistantship shows significant investment in her development as a researcher.

Weaknesses

- None noted

Study Timeline

Strengths

- Using the first year largely to take courses and improve technically is good for the PI’s technical abilities. However, if the field is developing as quickly as it seems, she might get scooped if she waits too long to dive into the research.

Weaknesses

- The project with the number of subjects indicated could likely be completed in less than five years.

Protections for Human Subjects

Acceptable Risks and Adequate Protections

- Very well done

Data and Safety Monitoring Plan (Applicable for Clinical Trials Only):

Acceptable

Inclusion Plans

- Sex/Gender: Distribution justified scientifically
- Race/Ethnicity: Distribution justified scientifically
- For NIH-Defined Phase III trials, Plans for valid design and analysis: Scientifically acceptable
- Inclusion/Exclusion Based on Age: Distribution justified scientifically

Biohazards

Acceptable

Training in the Responsible Conduct of Research

Acceptable

Resource Sharing Plans

Acceptable

Authentication of Key Biological and/or Chemical Resources

Acceptable

Budget and Period of Support

Recommend as Requested

CRITIQUE 3

Candidate: 2
Career Development Plan/Career Goals: 2
Research Plan: 3
Mentor(s), Co-Mentor(s), Consultant(s), Collaborator(s): 3
Environment Commitment to the Candidate: 2

Overall Impact: This is a proposal from a kinesiologist scientist who trained at the University of Kentucky and currently is an Assistant Professor at Michigan State University. The candidate will

acquire training in functional MRI, statistics, functional biomechanics, grantsmanship and professional development. The candidate clearly has the potential to build a research career as a physician scientist. The goal is to study the fear of reinjury using fMRI, as fear modulates landing patterns, and is one of the causes of reinjury in ACL reconstructions. The proposal aims to determine the role of Virtual Reality Mindfulness Meditation in reducing such fears, and thus reinjury, and ultimate pursue an R01 clinical trial to evaluate the role of VRMM in ACLR reinjuries over 24 months. The professional development and grantsmanship will be conducted via the USBJYII programs, and Dr. Pontifex is an excellent mentor with funding and experience in mentoring students and postdoctoral researchers. Dr. Liu who will provide mentorship in fMRI is also suitable renowned. Dr. Pietrosimone is at UNC and will provide mentorship of functional biomechanics; his being remote and providing mentorship in an area requiring hands on experimentation and data gathering is a matter of some concern. The goal is to study the impact of VRMM over 8 weeks on women 9 months post ACLR on (i) reducing self-reported injury related fear, (ii) jump landing biomechanics; and (iii) suppression of default mode networks in brain fMRI. The fundamental issue remains whether 8 weeks of VRMM will produce detectable changes on the DMN specifically in injury related fear. DMN is modified by VRMM changes in pain and other psychopathological populations but catastrophizing fear of pain seems to be far larger than fear of reinjury. The resources are appropriate.

THE FOLLOWING SECTIONS WERE PREPARED BY THE SCIENTIFIC REVIEW OFFICER TO SUMMARIZE THE OUTCOME OF DISCUSSIONS OF THE REVIEW COMMITTEE, OR REVIEWERS' WRITTEN CRITIQUES, ON THE FOLLOWING ISSUES:

PROTECTION OF HUMAN SUBJECTS: UNACCEPTABLE
The research plan includes children under the age of 18 and lacks parental informed consent. In addition, there is a concern that double-blind trial study safety monitoring will be done by the PI.

INCLUSION OF WOMEN PLAN: ACCEPTABLE

INCLUSION OF MINORITIES PLAN: ACCEPTABLE

INCLUSION ACROSS THE LIFESPAN: ACCEPTABLE

TRAINING IN THE RESPONSIBLE CONDUCT OF RESEARCH: ACCEPTABLE

COMMITTEE BUDGET RECOMMENDATIONS: The budget was recommended as requested.

Footnotes for 1 K23 AR079056-01; PI Name: Baez, Shelby

NIH has modified its policy regarding the receipt of resubmissions (amended applications).See Guide Notice NOT-OD-18-197 at https://grants.nih.gov/grants/guide/notice-files/NOT-OD-18-197.html. The impact/priority score is calculated after discussion of an application by averaging the overall scores (1-9) given by all voting reviewers on the committee and multiplying by 10. The criterion scores are submitted prior to the meeting by the individual reviewers assigned to an application, and are not discussed specifically at the review meeting or calculated into the overall impact score. Some applications also receive a percentile ranking. For details on the review process, see http://grants.nih.gov/grants/peer_review_process.htm#scoring.

MEETING ROSTER

Arthritis and Musculoskeletal and Skin Diseases Special Grants Review Committee
Arthritis and Musculoskeletal and Skin Diseases Initial Review Group
NATIONAL INSTITUTE OF ARTHRITIS AND MUSCULOSKELETAL AND SKIN DISEASES
AMS
10/22/2020 - 10/23/2020

Notice of NIH Policy to All Applicants: Meeting rosters are provided for information purposes only. Applicant investigators and institutional officials must not communicate directly with study section members about an application before or after the review. Failure to observe this policy will create a serious breach of integrity in the peer review process, and may lead to actions outlined in NOT-OD-14-073 at https://grants.nih.gov/grants/guide/notice-files/NOT-OD-14-073.html and NOT-OD-15-106 at https://grants.nih.gov/grants/guide/notice-files/NOT-OD-15-106.html, including removal of the application from immediate review.

CHAIRPERSON(S)

COSTENBADER, KAREN H, MD, MPH
PROFESSOR
DIVISION OF RHEUMATOLOGY
DEPARTMENT OF MEDICINE
BRIGHAM AND WOMEN'S HOSPITAL
BOSTON, MA 02115

MEMBERS

AGARWAL, SANDEEP K, BA, PHD, MD *
ASSOCIATE PROFESSOR
DEPARTMENT OF IMMUNOLOGY, ALLERGY,
AND RHEUMATOLOGY
BAYLOR COLLEGE OF MEDICINE
HOUSTON, TX 77030

BAAR, KEITH, PHD *
PROFESSOR
DEPARTMENT OF NEUROBIOLOGY
SCHOOL OF MEDICINE
UNIVERSITY OF CALIFORNIA DAVIS
DAVIS, CA 95616

BERONJA, SLOBODAN, BS, PHD *
ASSOCIATE PROFESSOR
HUMAN BIOLOGY DIVISION
FRED HUTCHINSON CANCER RESEARCH CENTER
SEATTLE, WA 98109

BEYNNON, BRUCE, PHD
MCCLURE PROFESSOR OF MUSCULOSKELETAL RESEARCH
AND DIRECTOR
DEPARTMENT OF ORTHOPEDICS AND REHABILITATION
MCCLURE MUSCULOSKELETAL RESEARCH CENTER
UNIVERSITY OF VERMONT LARNER COLLEGE OF MEDICINE
BURLINTON, VT 05405

BONEWALD, LYNDA F, PHD
PROFESSOR AND DIRECTOR
INDIANA CENTER FOR MUSCULOSKELETAL HEALTH
DEPARTMENTS OF ANATOMY, CELL BIOLOGY &
PHYSIOLOGY,
AND ORTHOPAEDIC SURGERY
INDIANA UNIVERSITY
INDIANAPOLIS, IN 46202

BOTCHWEY, EDWARD A, PHD
ASSOCIATE PROFESSOR
WALLACE H. COULTER DEPARTMENT OF
BIOMEDICAL ENGINEERING
GEORGIA INSTITUTE OF TECHNOLOGY
ATLANTA, GA 30307

BRUCKNER, ANNA L, MD
PROFESSOR OF DERMATOLOGY AND PEDIATRICS
UNIVERSITY OF COLORADO SCHOOL OF MEDICINE
SECTION HEAD, PEDIATRIC DERMATOLOGY
DIRECTOR, PEDIATRIC DERMATOLOGY FELLOWSHIP
CHILDREN’S HOSPITAL COLORADO
AURORA, CO 80045

CHANG, GREGORY, MD, AB *
ASSOCIATE PROFESSOR
MUSCULOSKELETAL IMAGING DIVISION
DEPARTMENT OF RADIOLOGY
NEW YORK UNIVERSITY LANGONE MEDICAL CENTER
NEW YORK, NY 10016

CHEN, ZHAO, MPH, PHD
PROFESSOR AND CHAIR
DEPARTMENT OF EPIDEMIOLOGY AND BIOSTATISTICS
MEL AND ENID ZUCKERMAN COLLEGE OF PUBLIC HEALTH
UNIVERSITY OF ARIZONA
TUCSON, AZ 85724

CRANE, JANET L, BS, MD *
ASSISTANT PROFESSOR
DIVISION OF ENDOCRINOLOGY
DEPARTMENT OF PEDIATRICS
DEPARTMENT OF ORTHOPAEDIC SURGERY
JOHNS HOPKINS UNIVERSITY
BALTIMORE, MD 21287

CROFFORD, LESLIE J, MD *
DIRECTOR AND PROFESSOR
DIVISION OF RHEUMATOLOGY
VANDERBUILT UNIVERSITY
NASHVILLE, TN 37232

DEMEHRI, SHADMEHR, BS, PHD, MD *
ASSISTANT PROFESSOR
DEPARTMENT OF DERMATOLOGY AND
HIGH RISK SKIN CANCER (ORGAN TRANSPLANTS)
MASSACHUSETTS GENERAL HOSPITAL
BOSTON, MA 02114

DIRKSEN, ROBERT T, PHD
LEWIS PRATT ROSS PROFESSOR AND CHAIR
DEPARTMENT OF PHARMACOLOGY AND PHYSIOLOGY
UNIVERSITY OF ROCHESTER
ROCHESTER, NY 14642

DUDLEY, ANDREW T, PHD *
ASSOCIATE PROFESSOR
HOLLAND REGENERATIVE MEDICINE PROGRAM
DEPARTMENT OF GENETICS,
CELL BIOLOGY AND ANATOMY
THE UNIVERSITY OF NEBRASKA MEDICAL CENTER
OMAHA, NE 608198

EVSEENKO, DENIS, MD, PHD *
ASSOCIATE PROFESSOR
ORTHOPAEDIC SURGERY
HEALTH SCIENCES CAMPUS
KECK SCHOOL OF MEDICINE
UNIVERSITY OF SOUTHERN CALIFORNIA
LOS ANGELES, CA 90033

FREEDMAN, TANYA S., AB, PHD *
ASSISTANT PROFESSOR
DEPARTMET OF PHARMACOLOGY
UNIVERSITY OF MINNESOTA
MINNEAPOLIS, MN 55455

GANESAN, ANAND K, PHD, MD
ASSOCIATE PROFESSOR
DEPARTMENT OF DERMATOLOGY
UNIVERSITY OF CALIFORNIA, IRVINE
IRVINE, CA 92697

JARJOUR, WAEL N, MD *
PROFESSOR
MARTHA MOREHOUSE CHAIR, ARTHRITIS
AND IMMUNOLOGY RESEARCH
DEPARTMENT OF INTERNAL MEDICINE
OHIO STATE UNIVERSITY
COLUMBUS, OH 43210

JONES, LYNNE C., PHD, MS, BS *
ASSOCIATE PROFESSOR
DEPARTMENT OF ORTHOPAEDIC SURGERY
JOHNS HOPKINS UNIVERSITY SCHOOL OF MEDICINE
BALTIMORE, MD 21224

KAMEN, DIANE L, MD, MSCR *
ASSOCIATE PROFESSOR OF MEDICINE
DIVISION OF RHEUMATOLOGY AND IMMUNOLOGY
MEDICAL UNIVERSITY OF SOUTH CAROLINA
CHARLESTON, SC 29425

KARDON, GABRIELLE, PHD
PROFESSOR
DEPARTMENT OF HUMAN GENETICS
UNIVERSITY OF UTAH
SALT LAKE CITY, UT 84112

KASHIKAR-ZUCK, SUSMITA, PHD
PROFESSOR
DIVISION OF BEHAVIORAL MEDICINE AND
CLINICAL PSYCHOLOGY
CINCINNATI CHILDREN'S HOSPITAL MEDICAL CENTER
CINCINNATI, OH 45229

KATES, STEPHEN LLOYD, MD, BS *
PROFESSOR AND CHAIR
THE JOHN CARDEA CHAIR OF ORTHOPAEDICS
DEPARTMENT OF ORTHOPAEDIC SURGERY
SCHOOL OF MEDICINE
VIRGINIA COMMONWEALTH UNIVERSITY
RICHMOND, VA 23219

KIM, BRIAN S, MD
ASSOCIATE PROFESSOR
DIVISION OF DERMATOLOGY
DEPARTMENT OF MEDICINE
WASHINGTON UNIVERSITY SCHOOL OF MEDICINE
ST. LOUIS, MO 63110

LANE, JOSEPH MICHAEL, MD
PROFESSOR AND ASSISTANT DEAN
DEPARTMENT OF ORTHOPAEDIC SURGERY
HOSPITAL FOR SPECIAL SURGERY
WEILL CORNELL MEDICINE
NEW YORK, NY 10021

LASSAR, ANDREW BRUCE, PHD, BA *
PROFESSOR
DEPARTMENT OF BIOLOGICAL CHEMISTRY
AND MOLECULAR PHARMACOLOGY
HARVARD MEDICAL SCHOOL
BOSTON, MA 02115

LOESER, RICHARD F, MD *
HERMAN AND LOUISE SMITH DISTINGUISHED PROFESSOR
DIRECTOR,THURSTON ARTHRITIS RESEARCH CENTER
UNIVERSITY OF NORTH CAROLINA AT CHAPEL HILL
CHAPEL HILL, NC 27599

MAJUMDAR, SHARMILA, PHD
PROFESSOR AND VICE CHAIR OF RESEARCH
DEPARTMENT OF RADIOLOGY AND BIOMEDICAL IMAGING
UNIVERSITY OF CALIFORNIA, SAN FRANCISCO
SAN FRANCISCO, CA 94158

O'CONNELL, GRACE D, BS, PHD *
ASSISTANT PROFESSOR
MECHANICAL ENGINEERING
UNIVERSITY OF CALIFORNIA BERKELEY
BERKELEY, CA 94720

OGDIE-BEATTY, ALEXIS R, MD
ASSOCIATE PROFESSOR
DIVISION OF RHEUMATOLOGY
DEPARTMENTS OF MEDICINE AND EPIDEMIOLOGY
PERELMAN SCHOOL OF MEDICINE
UNIVERSITY OF PENNSYLVANIA
PHILADELPHIA, PA 19106

PALMIERI-SMITH, RIANN MARIE, PHD, MS, ATC *
ASSOCIATE PROFESSOR
DEPARTMENT OF ORTHOPEDIC SURGERY
SCHOOL OF KINESIOLOGY
UNIVERSITY OF MICHIGAN
ANN ARBOR, MI 48109

SLOBOGEAN, GERARD, MD, BS, MPH, RESIDENCY *
ASSOCIATE PROFESSOR
ASSISTANT DIRECTOR OF CLINICAL RESEARCH FOR
THE DEPARTMENT OF ORTHOPAEDICS
UNIVERSITY OF MARYLAND
BALTIMORE, MD 21201

WILLIAMS, BART O, PHD *
PROFESSOR AND DIRECTOR
CENTER FOR CANCER AND CELL BIOLOGY
DIRECTOR, PROGRAM FOR SKELETAL DISEASE
AND TUMOR MICROENVIRONMENT
VAN ANDEL RESEARCH INSTITUTE
GRAND RAPIDS, MI 49503

XU, HUI, PHD, MD
PROFESSOR
DEPARTMENT OF DERMATOLOGY
UNIVERSITY OF ALABAMA AT BIRMINGHAM
BIRMINGHAM, AL 35294

YAMMANI, RAGHUNATHA R, PHD, BS *
ASSOCIATE PROFESSOR
INTERNAL MEDICINE-MOLECULAR MEDICINE
WAKE FOREST SCHOOL OF MEDICINE
WINSTON-SALEM, NC 27157

SCIENTIFIC REVIEW OFFICER

LIN, HELEN, PHD
SCIENTIFIC REVIEW OFFICER
NATIONAL INSTITUTE OF ARTHRITIS AND
MUSCULOSKELETAL AND SKIN DISEASES
NATIONAL INSTITUTES OF HEALTH
BETHESDA, MD 20892

EXTRAMURAL SUPPORT ASSISTANT

NGANDU, NAABA
EXTRAMURAL SUPPORT ASSISTANT
NATIONAL INSTITUTES OF HEALTH
NATIONAL INSTITUTE OF ARTHRITIS AND
MUSCULOSKELETAL AND SKIN DISEASES
BETHESDA, MD 20892

* Temporary Member. For grant applications, temporary members may participate in the entire meeting or may review only selected applications as needed.

Consultants are required to absent themselves from the room during the review of any application if their presence would constitute or appear to constitute a conflict of interest.
